# Cross-sectional relationships of circadian misalignment and rest-activity rhythms with occupational attainment in UK Biobank participants

**DOI:** 10.1080/07420528.2024.2441192

**Published:** 2024-12-23

**Authors:** Benjamin Walker, Jon Gibson, Callum Jackson, John Blaikley, Samuel E. Jones, Martin K. Rutter, Matt Sutton

**Affiliations:** a Division of Population Health, Health Services Research & Primary Care, The University of Manchester, Manchester, UK; b Department of Mathematics, University of Manchester, Manchester, UK; c Faculty of Biology, Medicine and Health, University of Manchester, Manchester, UK; d Wythenshawe Hospital, MFT, Manchester, UK; e Institute for Molecular Medicine Finland (FIMM), University of Helsinki, Helsinki, Finland; f Division of Diabetes, Endocrinology and Gastroenterology, School of Medical Sciences, Faculty of Biology, Medicine and Health, University of Manchester, Manchester, UK; g Diabetes, Endocrinology and Metabolism Centre, NIHR Manchester Biomedical Research Centre, Manchester University Hospitals NHS Foundation Trust, Manchester, UK

**Keywords:** Circadian misalignment, rest-activity rhythms, occupation, UK Biobank

## Abstract

Circadian misalignment and disrupted rest-activity rhythms have been linked to adverse health and educational outcomes, yet few studies have considered their relationships with economic outcomes. We investigate associations between multiple accelerometer-measured circadian misalignment traits (Composite Phase Deviation and the Sleep Regularity Index) and rest-activity rhythm traits (Inter-daily Stability, Intra-daily variability and relative amplitude), with occupational attainment, measured using the average wage paid to an individual’s occupation. We use data from 20 356 UK Biobank participants who wore an accelerometer (2013–16), provided employment data for the year they wore the accelerometer, and supplied covariate data at recruitment (2006–10). We use linear regression, with incremental adjustments for four sets of covariates, and stratify our analysis by sex. Our findings provide mixed evidence on the relationship between circadian misalignment and occupational attainment, varying by the measure of circadian misalignment used, and sex. We find fragmented rest-activity rhythms to be associated with higher occupational attainment, which is counterintuitive. Since circadian misalignment is a modifiable trait, our results suggest potential benefits of policies aimed at reducing circadian misalignment, such as altering work schedules and reducing bright light exposure in the evening. Further research is needed to elucidate the mechanisms through which rest-activity rhythms may impact economic outcomes.

## Introduction

We live in a 24-h environment to which our internal processes are aligned (Meléndez-Fernández et al. 2023; Roenneberg et al. 2019; Vetter 2018; Wittmann et al. 2006). However, modern-day environments including bright light exposure at night, 24-h availability of food and shift-work challenge this alignment and have detrimental effects.

Circadian misalignment can be defined as a mismatch between an individual’s internal biological clock (circadian rhythm), of which the sleep/wake cycle is a part, and the external environment or behavioural schedule the individual is living by. Misaligned circadian rhythms are widespread, affecting more than two-thirds of the general population (Wittmann et al. 2006). There is accumulating evidence of the far-reaching health and social impacts of circadian misalignment (Mason et al. 2020). Circadian misalignment is associated with shorter sleep duration and lower sleep quality (Baron and Reid 2014). Previous studies have found circadian misalignment to be linked to lower cognitive performance, higher BMI, harmful health behaviours, as well as many adverse physical and mental health outcomes, including prostate and breast cancer, type 2 diabetes, depression and anxiety (Baron and Reid 2014; Chellappa et al. 2018; Dickerman et al. 2016; Hansen and Stevens 2012; Kalmbach et al. 2015; Roenneberg et al. 2012; Schernhammer et al. 2001; Vetter et al. 2015; Wittmann et al. 2006).

Previous studies that look at the relationship between circadian misalignment and economic outcomes have tended to use social jetlag as the exposure. Social jetlag is a useful measure of circadian misalignment but does not take advantage of intra-individual day-to-day variation in sleep timing (Fischer et al. 2021). Novel metrics that take advantage of intra-individual day-to-day variation in sleep timing to capture circadian misalignment, including Composite Phase Deviation (CPD) and the Sleep Regularity Index (SRI) have been developed.

A related, but separate disorder to circadian misalignment is “rest-activity rhythm disruption.” Rest-activity rhythms refer to the “magnitude, timing and regularity of rest-activity patterns” (Li et al. 2021). These are a manifestation of the circadian organisation of the sleep-wake cycle (Baron and Reid 2014; Chellappa et al. 2018; Dickerman et al. 2016; Hansen and Stevens 2012; Kalmbach et al. 2015; Roenneberg et al. 2012; Schernhammer et al. 2001; Vetter et al. 2015; Wittmann et al. 2006). Indicators of disrupted (blunted) rest-activity rhythms, such as low day-to-day stability (inter-daily stability) and fragmented daily activity patterns (intra-daily variability), have been associated with adverse health outcomes such as Alzheimer’s and metabolic syndrome (Hoopes et al. 2021; Li et al. 2020).

Despite the growing evidence base establishing the impact of circadian misalignment on sleep, health and education, which are known to impact economic outcomes, there is a notable research gap concerning its association with economic outcomes. This is also true of disrupted rest-activity rhythms, which have been identified as a powerful predictor of adverse health outcomes such as mortality risk (Leroux et al. 2021; Xu et al. 2022; Zuurbier et al. 2015). Understanding how circadian misalignment and rest-activity rhythm disruption are associated with occupational attainment and earnings is important to assessing their potential broader socio-economic implications including work schedules.

There is an extensive literature describing relationship between sleep irregularity, dampened RAR, and adverse health outcomes including cognitive dysfunction (Dickerman et al. 2016; Hoopes et al. 2021; Roenneberg et al. 2012; Schernhammer et al. 2001; Zuurbier et al. 2015), and cognitive decline (Chellappa et al. 2018; Haghayegh et al. 2024; Hoopes et al. 2021; Luik et al. 2013; Zuurbier et al. 2015). With health and better cognitive skills, each being associated with better economic outcomes (Contoyannis and Rice 2001; Heckman et al. 2006; Lenhart 2019), we hypothesise that sleep irregularity, circadian misalignment and disturbed rest-activity rhythms will be associated with lower occupational attainment.

## Methods and Materials

### Data

We use data from 20 356 UK Biobank participants who were aged 40–69 y when they were recruited between 2006 and 2010 across 22 UK centres (Sudlow et al. 2015). NHS patient registers were used to recruit participants, with individuals being contacted if they lived close to one of the 22 centres. Data collected include demographic information, health behaviours including self-reported sleep traits, biological data, medical history, and economic and social information. Additional data on 122,495 participants were collected using an online follow-up work history questionnaire undertaken between 2013 and 2015 (Work history questionnaire 2022). Further data on physical activity and rest were collected using an Axivity A×3triaxial accelerometer delivered by mail and worn for up to seven consecutive days between 2013 and 2016.

**Figure 1.**
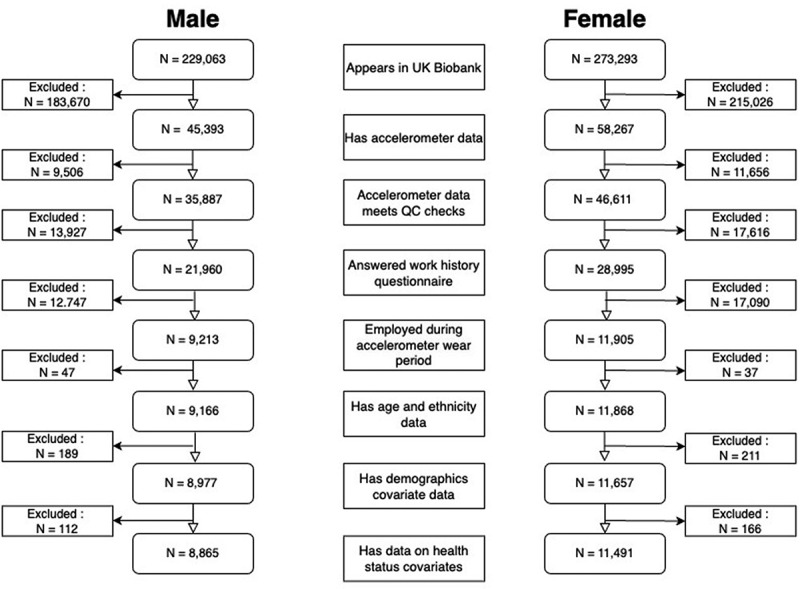
Study cohort construction flowchart.

A flowchart detailing how we arrived at our final sample of 8,865 men and 11 491 women is shown in Figure 1. The total cohort was 502,356 consisting of 229,063 men and 273,293 women. 183,670 men and 215,026 women do not have accelerometry data and therefore are excluded from the analysis leaving 45 393 men and 58 267 women. A further 9,506 men and 11 656 women are excluded as their accelerometry data did not fulfil quality control checks. Quality control checks ensured: no data problems, sufficient wear time, no data calibration issues, wear period not overlapping with daylight savings, no more than 1 data interruption, no more than 1 data recording error, sleep period not less than 3 h, number of sleep episodes per night being more than 5 and less than 30, average wake up time not in the afternoon, and 2 or more days of valid data. We lose 26 674 men and 34 706 women from the sample when we restrict to those responding to the work history questionnaire and are employed at the time the accelerometer was worn. Finally, we lose 348 men and 414 women who do not have data on all covariates, leaving us a final cohort of 8,865 men and 11 491 women.

### Exposures

We use two measures of circadian misalignment and three measures of rest-activity rhythm stability as exposures. These exposures were derived from accelerometer data using the open-source GGIR algorithm (Jones et al. 2019; van Hees et al. 2018).

(a) Composite phase deviation (CPD) is a composite measure of (i) how different the midsleep times are compared to those on the previous day, and (ii) how far away midsleep times occur from an individual’s preferred sleep timing (chronotype, as measured by midsleep time on weekends) (Fischer et al. 2016; O’Loughlin et al. 2021; Vetter 2018). This metric, developed by Fischer et al. (2016). builds on the concept of social jetlag by incorporating longitudinal day-to-day variation in sleep timing. Higher CPD (indicative of higher circadian misalignment) is linked to later chronotype (which is often associated with circadian misalignment), lower mood and well-being, as well as more variable calorific intake (Fischer et al. 2016, 2020, 2021; Imam et al. 2020).

Equation 1 shows how CPD is estimated:

(1) $$\mathrm{CPD}=\sqrt{F^{2}+Z^{2}}$$

in which F represents the difference between the time of mid-sleep on day *t* and the time of mid-sleep on a free-day (Friday night to Saturday morning, and Saturday night to Sunday morning). Z is the difference between the time of mid-sleep on day *t* and the time of mid-sleep on day *t*-1. From the accelerometer data, we get information on seven days of sleep, including two weekend days. We therefore take the mean of CPD over the 7-d period. On a free-day, F is equal to the difference in mid-sleep on that free-day from the mean of mid-sleep on both free-days.

(b) Sleep Regularity Index (SRI) is a measure of the regularity of an individual’s sleep pattern (Phillips et al. 2017). It equals 100 if an individual sleeps and wakes at the exact same time each day and equals 0 if sleep and wake times have no day-to-day overlap.

SRI is calculated by looking at the probability that someone is in the same sleep/wake state 24 h apart, and then calculating the proportion of time that the sleep patterns overlap. This can be done on a minute-by-minute basis. For example, seeing whether a cohort member was awake at 20:32 h, and then seeing if the same cohort member was awake at 20:32 h the next day (Lunsford-Avery et al. 2018).

(2) $$\mathrm{SR}I_{i}=-100+\frac{200}{M\left(N-1\right)}\overset{M}{\underset{j=1}{\sum }}\overset{N-1}{\underset{i=1}{\sum }}\delta \left(1\left(S_{\mathrm{\_t},j}=S_{\mathrm{\_t}+1,j}\right)\right)$$

Equation 2 taken from Lunsford-Avery et al. (2018), shows how SRI is calculated, where M is the number of datapoints in a day, and N is the number of days of data recorded. $S_{t,j}$ is binary and equals 0 if an individual is asleep and equals 1 if an individual is awake. The binary indicator of an individual being in the same sleep/wake state 24 h apart is 1($\delta \left(S_{t,j},S_{t+1,j}\right)$), which equals 1 if $S{\;}_{\_t,j}^{\;}=S_{\mathrm{\_t}+1,j}$ and equals 0 if not.

(c) Intra-daily variability (IV) measures the degree of fragmentation of the 24-h rest-activity rhythm by counting the total number of active and inactive periods. An alternative way of conceptualising intra-daily variability is that it measures the frequency with which individuals shift from rest to activity during a 24-h period (Zuurbier et al. 2015). Higher values indicate higher fragmentation of activity rhythms (Witting et al. 1990).

IV is measured using the ratio between successive datapoints (first-derivative) and the total variance of rest-activity across all observations. The method of calculating intra-daily variability is detailed in Equation 3.

(3) $$\mathrm{IV}=\frac{N\overset{N}{\underset{i=2}{\sum }}{\left(x_{i}-x_{i-1}\right)}^{2}}{\left(N-1\right)\overset{N}{\underset{i=1}{\sum }}{\left(x_{i}-\overset{ˉ}{x}\right)}^{2}}$$

where *N* is the total number of datapoints, *x*_i_ *i* represents the level of activity in epoch “i,” and $\overset{ˉ}{x}$ represents the mean level of activity across all datapoints.

(d) Relative Amplitude (RA) assesses the difference in average activity (as measured by acceleration), between the most active 10-h period of the day (M10), and the least active 5-h period of the day (L5) (Van Someren et al. 1999). A higher RA represents higher physical activity in the day, and a more restful sleep at night, and is therefore indicative of a more robust rest-activity rhythm. The formula for RA is detailed in equation 4 (Mitchell et al. 2017; Van Someren et al. 1999).

(4) $$\mathrm{RA}=\frac{M10-L5}{M10+L5}$$

(e) Inter-daily Stability (IS), a measure of the regularity of the rest-activity cycle (Vetter 2018). It quantifies how much each 24-h rest-activity profile overlaps with the next 24-h period, with higher values indicating greater overlap and greater day-to-day regularity (stability) of activity rhythms.

(5) $$\mathrm{IS}=\frac{N\overset{p}{\underset{h=1}{\sum }}{\left(\overset{ˉ}{x_{h}}-\overset{ˉ}{x}\right)}^{2}}{p\overset{N}{\underset{t=1}{\sum }}{\left(x_{t}-\overset{ˉ}{x}\right)}^{2}}$$

Equation 5 details how we estimate inter-daily stability (IS) which is computed by dividing the variance in activity across specific datapoints “h,” by the variance of rest-activity across all measured datapoints. Rest-activity data, collected through actigraphy, is divided into datapoints, which represent specific time intervals throughout the day. Each datapoints captures the activity level or lack thereof during that specific time frame. *x*_t_ represents the amount of rest-activity, measured using the amount of acceleration in datapoint “t,” $\overset{ˉ}{x_{h}}$represents the mean activity of datapoint “h” (a specific interval of the day) across all measured days and $\overset{ˉ}{x}$ represents the mean of all datapoint. N represents the total number of datapoints, and p represents the number of days in the measurement period (Fischer et al. 2021).

We z-score standardise all exposures by subtracting the sample mean and dividing by the SD to allow for comparison of the magnitude of association across different exposures. In order to help with interpretability, we reverse the signs (multiplied by −1) of Inter-daily Stability, Sleep Regularity Index, and Relative Amplitude, this means that our results show the association of a unit increase in unhealthy circadian misalignment, or rest-activity rhythms with occupational attainment.

We model each exposure independently, with multiple models considered for each exposure.

### Outcome

Our outcome variable is the natural logarithm of the average hourly wage paid to the occupation an individual was in at the time the accelerometer was worn (occupational attainment). We take the natural logarithm of occupational hourly wage to reduce the effect of outliers, and to approximate a normal distribution from right-skewed data.

We obtain our outcome by matching an individual’s occupation at the time the accelerometer was worn to the mean wage received by individuals in that occupation in the 2010 Annual Survey of Hours and Earnings (Office for National Statistics 2022).

Occupation is obtained from the job history questionnaire component of the online follow-up survey (2013–15) filled in by a sub-sample of Biobank participants. These responses were coded using the 4-digit Standard Occupational Classification 2000 (SOC-2000), which categorises occupations into 353 categories.

The 2010 Annual Survey of Hours and Earnings was conducted by the Office for National Statistics (Office for National Statistics 2022). We obtained the average hourly wage from Table 14.5a (Hourly pay – Gross 2010) of the 2010 Annual Survey of Hours and Earnings. Values are available for all occupations and can be matched to all 50 955 participants who provided accelerometer data and work history data.

We use this measure of occupational attainment because the UK Biobank did not collect data on participants’ earnings. This has some limitations, as it does not allow for variation in wages within specific occupational codes. Nonetheless, this approach has been used as a measure of occupational attainment or a substitute for wage in several previous papers (Bühler et al. 2020; Cobb-Clark and Tan 2011; Gordon et al. 2015; MacDonald and Pudney 2000; Macdonald and Shields 2001; McCollum et al. 2018; Morris 2006; Nickell 1982; Shields and Price 2002; van Ham and Manley 2015). It has been shown that average wages of four-digit SOC-Codes are predictive of actual wages, with an R^2^ of 0.488 (Clemens and Dibben 2014). Furthermore, four-digit SOC-Codes have been used to create synthetic measures of wages which correlate with health outcomes in similar ways to individual wages (Clemens and Dibben 2014).

### Stratification

Considering potential sex differences in labour market experiences and the potential varied effects of circadian misalignment on outcomes like education and obesity, we have stratified our analysis by sex (Díaz-Morales and Escribano 2015; Qian et al. 2019).

As a sensitivity analysis, to check that our results are not driven by employment schedules, such as shift work, which is linked to lower wages and may cause circadian misalignment, we have conducted additional analyses, restricted to full-time employees who do not work shifts (Figures 3 and 5).

**Figure 2.**
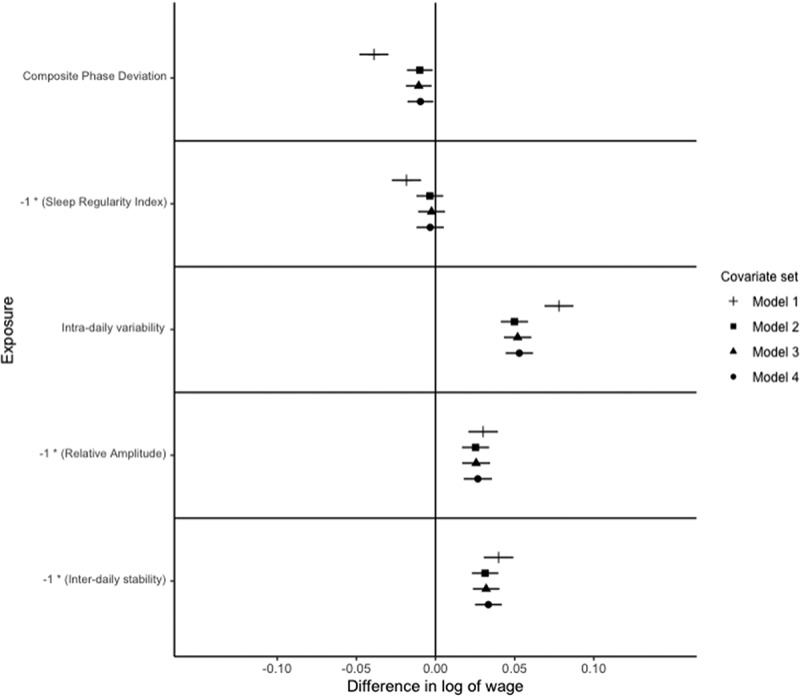
Relationships of circadian and rest-activity rhythms with a log of average occupational wage paid to occupation in males.

Notes: *N* = 8,865. The outcome is the difference in the log of occupational wage, which can be interpreted as the % difference in wage. Exposures are z-standardised for interpretability. Points represent point-estimate of the association between exposure and outcome, and bars represent 95% confidence intervals. The signs on Sleep Regularity Index, Relative Amplitude and Inter-daily stability are reversed for interpretability. Each exposure is modelled independently, with multiple models considered for each exposure. Model 1 adjusts for age, age squared and white ethnicity. Model 2 further adjusts for Government office region, urban vs. rural residence, educational attainment, marital status, child in the household, and shift work status, Model 3 adjusts for the number of related physical health conditions, depression, bipolar disorder, anxiety, BMI, alcohol consumption (categorised), smoking status, and vigorous physical activity status. Model 4 further adjusts for categorised accelerometer-derived sleep duration (<7 h, 7−9 h, and >9 h), and number of sleep episodes.

**Figure 3.**
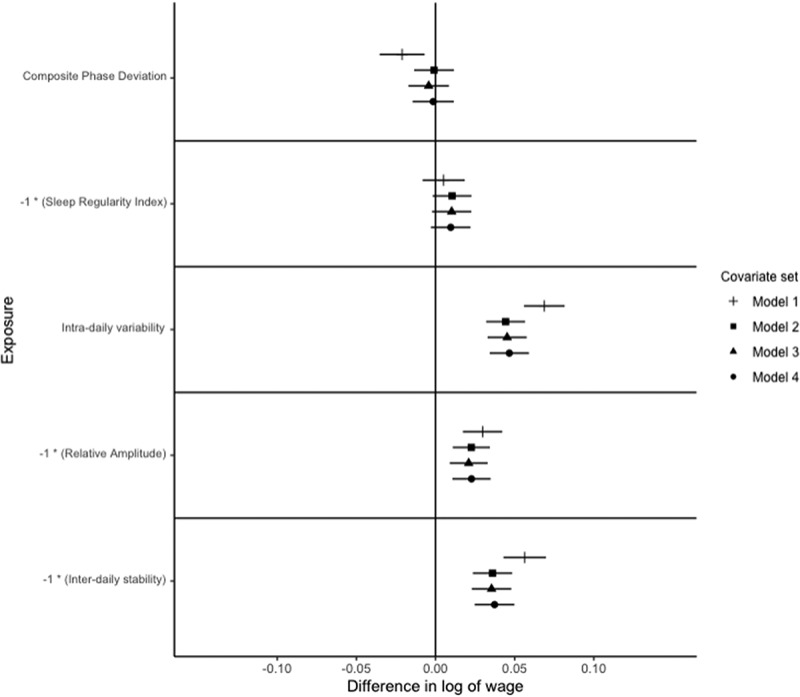
Relationships of circadian and rest-activity rhythms with a log of average occupational wage paid to occupation in non-shift working males in full-time employment.

Notes: *N* = 4,512. The outcome is the difference in the log of occupational wage, which can be interpreted as the % difference in wage. Exposures are z-standardised for interpretability. Points represent point-estimate of the association between exposure and outcome, and bars represent 95% confidence intervals. The signs on Sleep Regularity Index, Relative Amplitude and Inter-daily stability are reversed for interpretability. Each exposure is modelled independently, with multiple models considered for each exposure. Model 1 adjusts for age, age squared and white ethnicity. Model 2 further adjusts for Government office region, urban vs. rural residence, educational attainment, marital status, child in the household, and shift work status, Model 3 adjusts for the number of related physical health conditions, depression, bipolar disorder, anxiety, BMI, alcohol consumption (categorised), smoking status, and vigorous physical activity status. Model 4 further adjusts for categorised accelerometer-derived sleep duration (<7 h, 7−9 h, and >9 h), and number of sleep episodes. The sample is restricted to individuals who did not work shifts and worked full-time.

**Figure 4.**
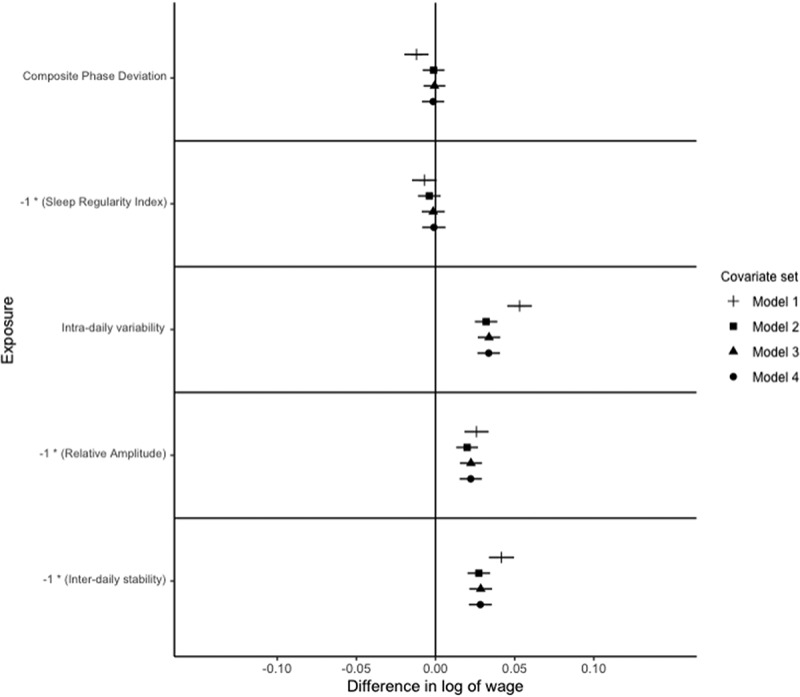
Relationships of circadian and rest-activity rhythms with a log of average occupational wage paid to occupation in females.

Notes: *N* = 11,492. The outcome is the difference in the log of occupational wage, which can be interpreted as the % difference in wage. Exposures are z-standardised for interpretability. Points represent point-estimate of the association between exposure and outcome, and bars represent 95% confidence intervals. The signs on Sleep Regularity Index, Relative Amplitude and Inter-daily stability are reversed for interpretability. Each exposure is modelled independently, with multiple models considered for each exposure. Model 1 adjusts for age, age squared and white ethnicity. Model 2 further adjusts for Government office region, urban vs. rural residence, educational attainment, marital status, child in the household, and shift work status, Model 3 adjusts for the number of related physical health conditions, depression, bipolar disorder, anxiety, BMI, alcohol consumption (categorised), smoking status, and vigorous physical activity status. Model 4 further adjusts for categorised accelerometer-derived sleep duration (<7 h, 7−9 h, and >9 h), and number of sleep episodes.

**Figure 5.**
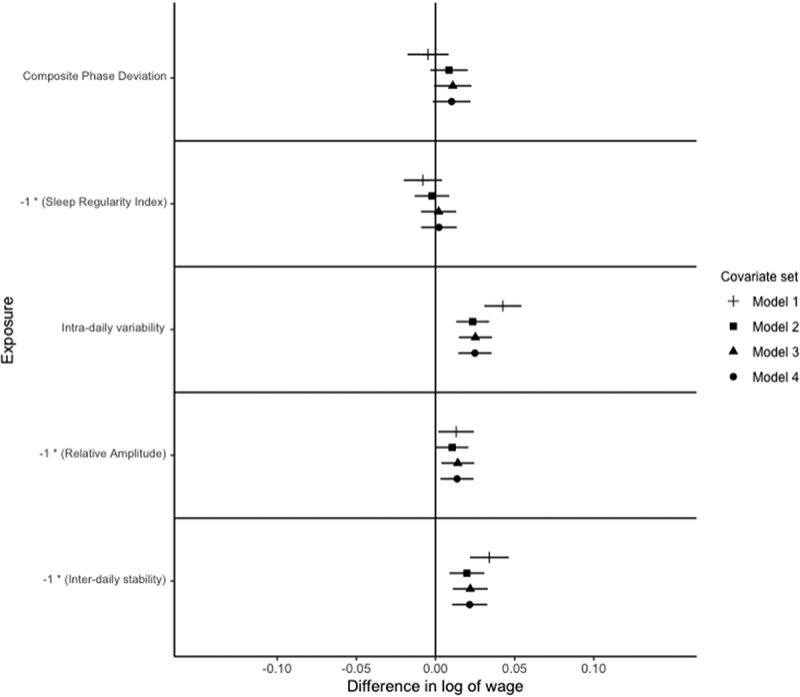
Relationships of circadian and rest-activity rhythms with a log of average occupational wage paid to occupation in non-shift working females in full-time employment.

Notes: *N* = 4,792. The outcome is the difference in the log of occupational wage, which can be interpreted as the % difference in wage. Exposures are z-standardised for interpretability. Points represent point-estimate of the association between exposure and outcome, and bars represent 95% confidence intervals. The signs on Sleep Regularity Index, Relative Amplitude and Inter-daily stability are reversed for interpretability. Each exposure is modelled independently, with multiple models considered for each exposure. Model 1 adjusts for age, age squared and white ethnicity. Model 2 further adjusts for Government office region, urban vs. rural residence, educational attainment, marital status, child in the household, and shift work status, Model 3 adjusts for the number of related physical health conditions, depression, bipolar disorder, anxiety, BMI, alcohol consumption (categorised), smoking status, and vigorous physical activity status. Model 4 further adjusts for categorised accelerometer-derived sleep duration (<7 h, 7−9 h, and >9 h), and number of sleep episodes. Sample is restricted to individuals who did not work shifts and worked full-time.

### Covariates

We adjust for four sets of covariates, the first set including confounders that cannot be on the causal pathway between circadian misalignment and rest-activity rhythm fragmentation with occupational attainment, and then add covariates that may be confounders or potential mediators of these relationships:

*Model 1 (Basic covariates)*: *Age and ethnicity* (binary variable for white/not white due to low numbers of non-white individuals).

*Model 2 (Demographics)*: Model 1 covariates plus: *Government office region, urban vs. rural residence, educational attainment, marital status, child in the household, and shift work status.*

*Model 3*: *(Health): number of related physical health conditions*,^1^ *depression, bipolar disorder, anxiety, BMI, alcohol consumption (categorised), smoking status, and vigorous physical activity status.*

*Model 4 (Sleep characteristics)*: model 3 covariates plus: *categorised accelerometer-derived sleep duration (<7 h, 7–9 h, and >9 h), and number of sleep episodes.*

### Empirical Analysis

We use Ordinary Least Squares (OLS) regression to estimate the effect of circadian misalignment on occupation attainment:

(6) Yi=τCi+BjXi+ui

in which Y is the log of average hourly wage in the individuals’ current occupation, and Ci represents our circadian misalignment exposure. Only one exposure is included in the regression at a time. X_i_ represents a vector of covariates, and u_i_ represents the residual. Individuals are indexed with the term “i.” *r* represents our parameter of interest; the association between our circadian misalignment exposure and log of average wage paid to an individual’s occupation. This can be interpreted as a percentage difference in the average wage paid to an individual’s occupation associated with a one-unit (equal to a standard deviation) change in exposure. We use robust-standard errors to address the issue of heteroscedasticity.

## Results

### Descriptive Statistics

Table S1 shows compares clinical characteristics of UK Biobank participants in our study sample, and those not included. Those included are observed to be more highly educated, younger, more physically active, and less likely to smoke (Table S1).

Table S2 shows summary statistics for occupational wage and our circadian misalignment and rest-activity stability exposures by one-digit Standard Occupational Classification. Compared to those in jobs that were classified as “Managers and Senior Officials” (SOC-Code 1), those with jobs classed as “elementary occupations” (SOC-Code 9) had lower occupational wage, lower intra-daily variability, higher inter-daily stability, higher composite phase deviation, lower sleep regularity index, and slightly higher relative amplitude, which is in line with results from our regression analysis (Table S2). Table S3 shows summary statistics for those who provided accelerometer data, but those are excluded due to not being in employment at the time they wore the accelerometer. Those excluded due to not being in work at the time they wore the accelerometer are on average older than our sample (mean age: 66 vs. 57) but have similar mean inter-daily stability, intra-daily variability and relative amplitude to our sample (Table S3).

A plot showing all possible values of average wage paid to occupation for the 353 categories of occupation, using data taken from the 2010 Annual Survey of Hours and Earning, can be found in Figure S1. A histogram showing the distribution of average wage paid to occupation in our sample can be found in Figure S2.

The mean age of participants at the time the accelerometer is worn is 57.3 y for males and 56.1 for females (Table 1). Just over half of our sample are educated to a degree level or higher (52% of males and females). Most of our sample are cohabiting with a partner at the time of recruitment (81% of males and 70% of females), 54% of men and 56% of women lived with a son or daughter at recruitment. In our sample, 96.8% of males and 96.7% of females are white. Most did at least 10 or more minutes of vigorous physical activity every week at recruitment (72% of men and 66% of women). 96% of men and 94.6% of women in our sample consume alcohol at least some of the time, while only 8% of men and 6% of women reported smoking. A small portion reported depression (2.8% of men and 5.4% of women).

**Table 1.** Clinical characteristics of men and women with actigraphy and derived occupational wage data.

	Men (*N* = 8,865)		Women (*N* = 11,492)	
Variable	Mean/%	Std. Dev.	Mean/%	Std. Dev.
Mean wage of occupation (£)	19.89	10.24	17.51	8.82
Intra-daily variability	0.66	0.19	0.64	0.17
Inter-daily stability (1=Perfect)	0.62	0.13	0.65	0.12
Sleep regularity index (100=Perfect)	58.5	11.67	62.9	11.00
Composite phase deviation	1.40	1.003	1.31	0.89
Highest qualification				
No qualification	3.6%		2.4%	
GCSEs or equivalent	11.1%		13.9%	
A Levels/AS Levels or equivalent	5.8%		7.3%	
Other professional qualification	8.9%		10.9%	
NVQ/HNC or equivalent	19.0%		13.4%	
Degree or higher	51.6%		52.0%	
White ethnicity	96.8%		96.7%	
Age (years)	57.3	6.6	56.1	5.8
Lives in urban area	85.4%		85.7%	
Shift-work				
No shift-work, %	87.0%		90.6%	
Shift-work but no night-shifts, %	2.6%		2.7%	
Night-shift work, %	10.4%		6.6%	
Married/cohabiting with partner	81.0%		69.7%	
Child	53.8%		56.3%	
Count of chronic physical illnesses	0.4	0.7	0.4	0.7
Depression	2.8%		5.4%	
Anxiety	0.9%		1.1%	
Bipolar	0.2%		0.2%	
BMI	27.1	4.0	26.0	4.9
Alcohol frequency				
Daily or almost daily	25.0%		16.2%	
3–4 times per week	28.9%		25.2%	
1–2 times per week	26.9%		27.4%	
1–3 times per month	9.7%		14.3%	
Special occasions only	5.5%		11.6%	
Never	4.0%		5.4%	
Smokes	8.2%		6.7%	
Does vigorous physical activity	72.1%		65.9%	

Prior to z-standardisation (subtracting the sample mean and dividing by sample S.D.) of CPD, males have a mean CPD of 1.4, females have a mean CPD of 1.3, and males have a mean intra-daily variability of 0.7 and females have a mean intra-daily variability of 0.6. The mean inter-daily stability is 0.6 for males and 0.7 for females. Males have a mean SRI of 58.5 and females have a mean SRI of 62.9.

The mean occupational hourly wage for males is £19.89, and for females is £17.51.

### Regression Analysis

#### Circadian Misalignment Exposures

##### Composite Phase Deviation

For men, after adjusting for model 1 (basic) covariates, a one SD increase in CPD is associated with a 3.9% [95% C.I.: 3.0%, 4.8%] lower average wage paid to occupation. After adjusting for model 2 (demographic) covariates, an S.D. increase in CPD is associated with a 1.0% [95% C.I.: 0.2%, 1.8%] lower average wage paid to the occupation (Figure 2). Adjusting for model 3 (health) covariates, a S.D. increase in CPD is associated with a 1.1% [95% C.I.: 0.2%, 1.9%] lower average wage paid to occupation, adjusting for model 4 (sleep) covariates attenuates the association to a 0.9% [95% C.I.: 0.1%, 1.8%] lower average wage paid to occupation (Figure 2). After restricting the sample to those who work full-time and do not work shifts, this relationship is only statistically significant for model 1 (Figure 3).

For women, after adjusting for model 1 covariates, an S.D. increase in CPD is associated with a 1.2% [95% C.I.: 0.4%, 2.0%] lower average wage paid to occupation, adjusting for model 2, 3 or 4 covariates attenuates this relationship to the null (Figure 4).

##### Sleep Regularity Index

For men, adjusting for model 1 covariates, a standard deviation lower SRI is associated with a 1.8% lower average wage paid to occupation [95% C.I.: 0.9%, 2.8%], adjusting for model 2, 3 or 4 covariates attenuates this to the null (Figure 2). After restricting the sample to full-time employed, non-shift workers, the relationship between SRI and occupational attainment is not significant for all models for men (Figure 3). For women, there is no significant relationship between SRI and occupational attainment, after adjusting for any set of covariates (Figure 4).

#### Rest-Activity Rhythm Exposures

##### Intra-Daily Variability

For men, after adjusting for model 1 covariates, a standard deviation higher IV is associated with a 7.8% [95% C.I.: 6.9%, 8.7%] higher average wage paid to occupation, adjusting for model 2 covariates attenuates this to a 5.0% [95% C.I.: 4.1%, 5.8%] higher average wage paid to occupation (Figure 2). Adjusting for model 3 covariates increases the magnitude of the relationship to a 5.2% [95% C.I.: 4.3%, 6.0%] higher average wage paid to occupation, adjusting for model 4 covariates further increases the magnitude to a 5.3% [95% C.I.: 4.4%, 6.2%] higher average wage paid to occupation (Figure 2).

After adjusting for model 1 covariates, a standard deviation higher IV is associated with a 5.3% [95% C.I.: 4.5%, 6.1%] higher average wage paid to occupation for women. Adjusting for model 2 covariates attenuates this to a 3.2% [95% C.I.: 2.5%, 3.9%] higher average wage paid to occupation (Figure 4). After adjusting for model 3 covariates, a standard deviation higher IV is associated with a 3.4% [95% C.I.: 2.7%, 4.1%] higher average wage paid to occupation, this is unchanged by adjusting for model 4 covariates (Figure 4).

For both men and women, findings are robust to the restriction of the sample to full-time employed, non-shift workers (Figures 3 and 5).

##### Relative Amplitude

After adjusting for model 1 covariates, an S.D. lower relative amplitude is associated with a 3.0% [95% C.I.: 2.1%, 3.9%] higher average wage paid to occupation for men (Figure 2). Adjusting for model 2 or 3 covariates, this association is attenuated to 2.5% [95% C.I.: 1.7%, 3.4%] higher average wage paid to occupation (Figure 2). The magnitude of this association is slightly increased to 2.7% [95% C.I.:1.8%, 3.6%] higher average wage paid to occupation after adjusting for model 4 covariates (Figure 2). These findings are robust to the restriction of the sample to full-time employed, non-shift workers (Figure 3).

For women, an S.D. lower relative amplitude is associated with a 2.6% [95% C.I.: 1.8%, 3.4%] higher average wage paid to occupation after model 1 covariates are adjusted for (Figure 4). Adjusting for model 2 covariates, this association is attenuated to 2.0% [95% C.I.: 1.3%, 2.7%]. After adjusting for model 3 or 4 covariates, the magnitude of the association increases to a 2.2% [95% C.I.: 1.5%, 2.9%] higher average wage paid to occupation. The associations between RA and occupational attainment in women are robust to the restriction of the sample to full-time employed, non-shift workers (Figure 5).

##### Inter-Daily Stability

F or men, an S.D. lower IS values were associated with a 4.0% [95% C.I.: 3.0%, 4.9%] higher average wage paid to occupation after adjusting for model 1 covariates; this is attenuated to a 3.1% [95% C.I.: 2.3%, 4.0%] higher average wage paid to occupation after adjusting for model 2 covariates (Figure 2). After adjusting for model 3 covariates, a S.D. higher IS is associated with a 3.2% [95% C.I.: 2.4%, 4.0%] higher average wage paid to occupation, and after adjusting for model 4 covariates the magnitude of this relationship is a 3.3% [95% C.I.: 2.5%, 4.2%] higher average wage paid to occupation (Figure 2).

For women, after adjusting for model 1 covariates, an S.D. lower IS is associated with a 4.2% [95% C.I.: 3.4%, 5.0%] higher average wage paid to occupation. After adjusting for model 2 covariates, an S.D. lower IS is associated with a 2.9% [95% C.I.: 2.1%, 3.6%] higher average wage paid to the occupation (Figure 3). Adjusting for model 3 covariates, an S.D. lower IS is associated with a 2.9% [95% C.I.: 2.1%, 3.6%] higher average wage paid to the occupation (Figure 4). Adjusting for model 4 covariates, an S.D. lower IS is associated with a 2.8% [95% C.I.: 2.1%, 3.6%] (Figure 4).

For both men and women, results are robust to restricting to full-time, non-shift workers (Figures 3 and 5).

## Discussion

### Overview and Interpretations of Findings

This study examines the relationship between rest-activity rhythm disruption and circadian misalignment with occupational attainment using regression methods adjusting for four sets of covariates. Regarding circadian misalignment, after full covariate adjustment, we find that higher CPD is associated with lower occupational attainment but only for men, restricting our sample to full-time employed, non-shift workers attenuates these associations to the null. We find no association between SRI and occupational attainment after full covariate adjustment.

Contrary to our initial hypothesis, we find that lower IS (greater variability in activity patterns across multiple days) and higher IV (greater fragmentation of activity during each day), which are indicative of disturbed rest-activity rhythms, are associated with higher occupational attainment. It is possible that disrupted rest-activity rhythms, while associated with higher occupational attainment, could lead to lower total earnings.

It is possible that disrupted rest-activity rhythms, while associated with higher occupational attainment, could lead to lower total earnings. This may occur because such disruptions are driven by demanding, higher-paying jobs that limit the hours worked. However, our sensitivity analyses (Figures 3 and 5) demonstrate that our findings remain robust when restricting the sample to individuals who were working full-time and not engaged in shift work.

The cross-sectional nature of our study means that we can only speculate on the direction of potential causal relationships, and the influence of shift work status. However, it seems feasible that shift work could confound relationships between dampened RAR and sleep irregularity with occupational attainment, since shift-work may cause dampened RAR and sleep irregularity, and be associated with jobs with lower average wages. However, results from our stratified analysis (Figures 3 and 5) show that our findings are robust to stratification to non-shift workers, suggesting that confounding by shift work status is not driving our findings.

Shift-work status could also moderate (interact with) relationships between dampened RAR and sleep irregularity with occupational attainment. Those who are prone to dampened RARs and/or sleep irregularity may struggle to work normal hours (9−5 PM), and shift work may allow them to work more effectively, and ultimately achieve higher occupational attainment.

Alternatively, shift work status may play a mediating role in these relationships since those with dampened RAR and sleep irregularity may select themselves into shift-working jobs which offer lower wages.

In interpreting our results, it is worth considering that whilst covariates in models 2 and 3, such as education, may be confounders, they may also be on the causal pathway linking circadian misalignment and rest-activity rhythms with occupational attainment. Therefore, when quantifying the strength and significance of relationships, model 1 might be considered more informative.

### Comparisons to Previous Literature

#### Circadian Misalignment

Our findings of no relationship between SRI and occupational attainment after full covariate adjustment is consistent with previous literature on the relationship between circadian misalignment (measured using social jetlag) and productivity (another economic outcome) (Ishibashi and Shimura 2020; Itani et al. 2022; Okajima et al. 2021; Takano et al. 2022). Our work builds on these prior studies which use self-reported social jetlag by our study using accelerometer-derived circadian misalignment measures, which reduces the risk of our results being influenced by measurement error and reporting bias (Ishibashi and Shimura 2020; Itani et al. 2022; Okajima et al. 2021; Takano et al. 2022). Furthermore, we build on prior studies by using a different outcome measure in occupational attainment, providing evidence on the relationship between circadian misalignment and different economic outcomes.

Our finding of CPD having a negative association with occupational attainment in men is in line with the findings of previous studies that find having an evening chronotype, which can be viewed as a proxy for circadian misalignment under the assumption evening chronotypes are more misaligned than morning chronotypes due to daytime commitments, is associated with lower earnings. These previous studies make use of large, representative cohorts, and have direct measures of wages (Bonke 2012; Conlin et al. 2023). Our work extends the work by Bonke (2012) and Conlin et al. (2023) by using a direct measure of circadian misalignment. Using chronotype as a proxy for circadian misalignment can lead to misclassification due to different individuals having different work-start times. Also, bias may be introduced by individuals selecting occupations in which work-start times suit their chronotype (Vetter 2018).

#### Rest-Activity Rhythms

Our findings that disturbed rest-activity rhythms as measured by lower relative amplitude, lower inter-daily stability and higher intra-daily variability are associated with higher occupational attainment, disagree with Mitchell et al. (2017), who found no association between rest-activity rhythms and income. Our study builds on this work by using a considerably larger, less selected sample.

### Strengths and Limitations

Our work has several strengths. To the best of our knowledge, this is the first study to assess relationships between direct measures of circadian misalignment (as opposed to simply using proxies such as chronotype or shift work) and rest-activity rhythms with economic outcomes. We are also able to use objectively assessed measures of circadian misalignment, which reduces the risk of our results being affected by measurement error. Our measures of circadian misalignment (SRI and CPD) allow for the capture of day-to-day instability in circadian rhythmicity, and also capture the biological impact of misalignment (Fischer et al. 2021). Furthermore, because of the rich nature of the dataset, we can control for multiple potential confounders including social, economic and health variables as well as sleep characteristics.

Our work also has several limitations. First, our data is cross-sectional, and therefore causal inference is not possible. As in all observational studies, our findings may be more prone to reverse causality and unmeasured confounding. Furthermore, due to the lack of a suitable instrumental variable, or unconfounded assignment of circadian misalignment and rest-activity rhythm stability, it is not possible for this study to implement quasi-experimental methods, which would allow for the estimation of causal relationships. Our cross-sectional study design means that we should be cautious about attributing causality when interpreting our observed associations between unstable rest-activity rhythms and being in a higher-paying occupation.

The UK Biobank is also not representative of the UK population, with its cohort members generally being older, white, and have higher socioeconomic status than the general population. This lack of representativeness could reduce the generalisability of the study’s findings to other populations. However, some studies (Batty et al. 2020; Gregson et al. 2019; Liu et al. 2023; Warrington et al. 2019) but not all (Keyes and Westreich 2019; Swanson 2012; Van Alten et al. 2022) have suggested that risk factor associations identified in the UK Biobank are generalisable. Furthermore, generalisability may be affected by attrition of the sample caused by limiting to UK Biobank participants who have data on relevant exposures, covariates and outcomes.

Furthermore, the cross-sectional design of our study means that our results could be influenced by survivor bias, such that individuals with demanding jobs, circadian misalignment and/or sleep disturbance could have become unwell or die and exclude themselves from participating.

### Future Research

Future research should evaluate the relationship between circadian misalignment and rest-activity rhythm disruption with occupational attainment using other methods, including prospective, or quasi-experimental methods to assess if our results are driven by unmeasured confounding or reverse causality. Future research should also examine the relationship between circadian misalignment and rest-activity rhythm disruption with other economic outcomes.

Future research should also seek to understand the mechanisms through which rest-activity rhythm disruption can impact economic outcomes.

Future studies should also seek to replicate our findings in cohorts that are more representative of the general population.

### Implications

Though it is currently unclear if rest-activity disruption is a modifiable trait (Jeon et al. 2023), circadian misalignment is a modifiable trait, which means there are opportunities for interventions if a causal relationship between circadian misalignment and occupational attainment can be confirmed in future research. For example, altering work schedules and reducing bright light exposure in the evenings are potential interventions. There are also potential opportunities for personalised interventions that modify work routines based on an individual’s natural sleep/wake time.

## Conclusion

This study examines the link between circadian misalignment and rest-activity rhythm disruption with wages using two accelerometer-derived measures of circadian misalignment, and three accelerometer-derived measures of rest-activity rhythm disruption. We find that circadian misalignment as measured by CPD is associated with lower occupational attainment and that a less stable rest-activity rhythm is associated with higher occupational attainment.

## Supplementary Material

Supplemental Material

## Note

1. Physical health conditions include: asthma, cancer, hypertension, stroke, diabetes, rheumatoid arthritis, hypothyroidism, heart failure, Chronic obstructive pulmonary disease (COPD), epilepsy, and atrial fibrillation.
