# Supplementary material for: Cross-sectional relationships of circadian misalignment and rest-activity rhythms with occupational attainment in UK Biobank participants
Source: Chronobiol Int. 2024 Dec 23;42(1):14–28. doi: 10.1080/07420528.2024.2441192 (PMC11854055; doi:10.1080/07420528.2024.2441192)
Supplement: Supplemental Material [file ICBI_A_2441192_SM7963.pdf]

Supplementary material

Table S1: Difference in characteristics between study sample and those not in sample

	In sample (N=20,356)			Out of sample (N=482,000)		
**Variable**	**Mean/Frequency**	**S.D/%**	**Observations**	**Mean/Frequency**	**S.D/%**	**Observations**
Male		43.5%	20,356		45.7%	482,000
Highest qualification			20,356			471,871
No qualification	604	3%		84,650	17.90%	
GCSEs or equivalent	2,577	12.7%		80,696	17.10%	
A/AS Levels or equivalent	1,360	6.7%		25,865	5.50%	
Other professional qualification	2,038	10%		57,148	12.10%	
NVQ/HNC or equivalent	3,223	15.8%		72,966	15.50%	
Degree or higher	10,554	51.8%		150,546	31.90%	
White ethnicity		96.7%	20,356		94.5%	479,224
Age at recruitment	50.5	6.2	20,356	56.8	8.1	482,000
Married/cohabiting with partner		74.6%	20,356		72.2%	482,001
Child in household		55.2%	20,356		33.9%	482,001
Count of chronic physical illnesses	0.4	0.7	20,356	0.7	0.9	482,001
Smokes		7.4%	20,356		10.7%	479,052
Does vigorous physical activity		68.6%	20,356		62.2%	454,428

Table S2: Means and SDs of outcomes and exposure by major occupational categories (1-digit SOC)

	Occupational wage		Intra-daily variability		Interdaily stability		CPD		SRI		Relative Amplitude	
	Male	Female	Male	Female	Male	Female	Male	Female	Male	Female	Male	Female
Managers and senior officials (SOC-CODE = 1)	29.96	27.88	0.68	0.65	0.61	0.64	1.32	1.32	59.07	62.93	0.84	0.85
(N-Men =1850) (N-Women =1597)	(14.85)	(13.87)	(0.19)	(0.17)	(0.12)	(0.12)	(0.85)	(0.87)	(11.14)	(10.83)	(0.08)	(0.07)
Professional occupations (SOC-CODE = 2)	22.21	22.11	0.70	0.65	0.61	0.64	1.30	1.25	59.09	63.56	0.85	0.86
(N-Men =3055) (N-Women =3355)	(4.90)	(5.43)	(0.18)	(0.17)	(0.12)	(0.11)	(0.87)	(0.79)	(11.50)	(10.65)	(0.08)	(0.07)
Associate professional and technical occupations (SOC-CODE = 3)	17.02	16.09	0.68	0.66	0.60	0.63	1.44	1.35	57.53	62.20	0.85	0.85
(N-Men =1610) (N-Women =2695)	(4.62)	(2.69)	(0.19)	(0.17)	(0.12)	(0.12)	(1.07)	(0.99)	(11.54)	(11.34)	(0.07)	(0.07)
Administrative and secretarial occupations (SOC-CODE = 4)	11.71	11.30	0.69	0.64	0.61	0.65	1.35	1.31	58.22	63.05	0.85	0.86
(N-Men =488) (N-Women =2215)	(1.20)	(1.31)	(0.18)	(0.16)	(0.12)	(0.11)	(0.85)	(0.84)	(11.62)	(10.68)	(0.07)	(0.07)
Skilled trades occupations (SOC-CODE = 5)	11.95	9.23	0.56	0.53	0.67	0.71	1.47	1.23	59.12	64.82	0.87	0.88
(N-Men =832) (N-Women =162)	(1.78)	(1.33)	(0.18)	(0.16)	(0.13)	(0.11)	(0.96)	(0.66)	(11.82)	(11.77)	(0.06)	(0.06)
Personal service occupations (SOC-CODE = 6)	9.55	8.88	0.60	0.59	0.65	0.67	1.49	1.35	57.78	63.25	0.86	0.87
(N-Men =169) (N-Women =820)	(1.50)	(1.08)	(0.16)	(0.17)	(0.13)	(0.13)	(1.26)	(1.07)	(11.32)	(11.88)	(0.07)	(0.07)
Sales and customer service occupations (SOC-CODE = 7)	9.48	8.31	0.62	0.55	0.64	0.67	1.60	1.44	56.79	61.45	0.85	0.87
(N-Men =147) (N-Women =356)	(1.32)	(1.17)	(0.20)	(0.16)	(0.13)	(0.11)	(1.42)	(0.95)	(12.90)	(10.73)	(0.08)	(0.06)
Process, plant and machine operatives (SOC-CODE = 8)	10.50	10.02	0.56	0.54	0.60	0.68	1.89	1.45	56.16	61.84	0.85	0.86
(N-Men =436) (N-Women =71)	(1.52)	(1.54)	(0.19)	(0.21)	(0.16)	(0.14)	(1.52)	(0.92)	(13.26)	(13.23)	(0.08)	(0.08)
Elementary occupations (SOC-CODE = 9)	8.70	7.52	0.58	0.58	0.63	0.66	1.77	1.50	55.83	61.21	0.86	0.87
(N-Men =278) (N-Women =220)	(0.97)	(0.86)	(0.20)	(0.19)	(0.14)	(0.14)	(1.40)	(1.09)	(13.05)	(11.19)	(0.07)	(0.07)

Table S3: Comparison of descriptive statistics of outcomes, and age between our sample and those who were excluded due to not being employed while accelerometer was worn

	Excluded due to not being employed while accelerometer was worn			Sample		
**Variable**	**Mean/Frequency**	**SD/%**	**Observations**	**Mean/Frequency**	**SD/%**	**Observations**
Male	0.427	0.495	29837	0.435	0.496	20356
Age (continuous)	66.6	5.548	29805	56.6	6.205	20356
Age (categorised)			29805			20356
49 or under	364	1.20%		3298	16.20%	
50-54	824	2.80%		5294	26.00%	
55-59	2094	7.00%		5696	28.00%	
60-64	6397	21.50%		4020	19.70%	
65-69	11530	38.70%		1584	7.80%	
70-74	7548	25.30%		422	2.10%	
75+	1048	3.50%		42	0.20%	
Intra-daily variability	0.66	0.182	29837	0.65	0.18	20356
Inter-daily stability	0.68	0.113	29837	0.63	0.12	20356
CPD	1.261	0.916	29837	1.35	0.94	20356
SRI	60.375	11.977	29837	60.98	11.51	20356
Relative Amplitude	0.853	0.069	29837	0.85	0.07	20356
Occupational wage at baseline	17.83	7.25	15736	17.88	7.4	19593

Figure S1: Plot of gross hourly wage paid to occupations (from ASHE 2014)

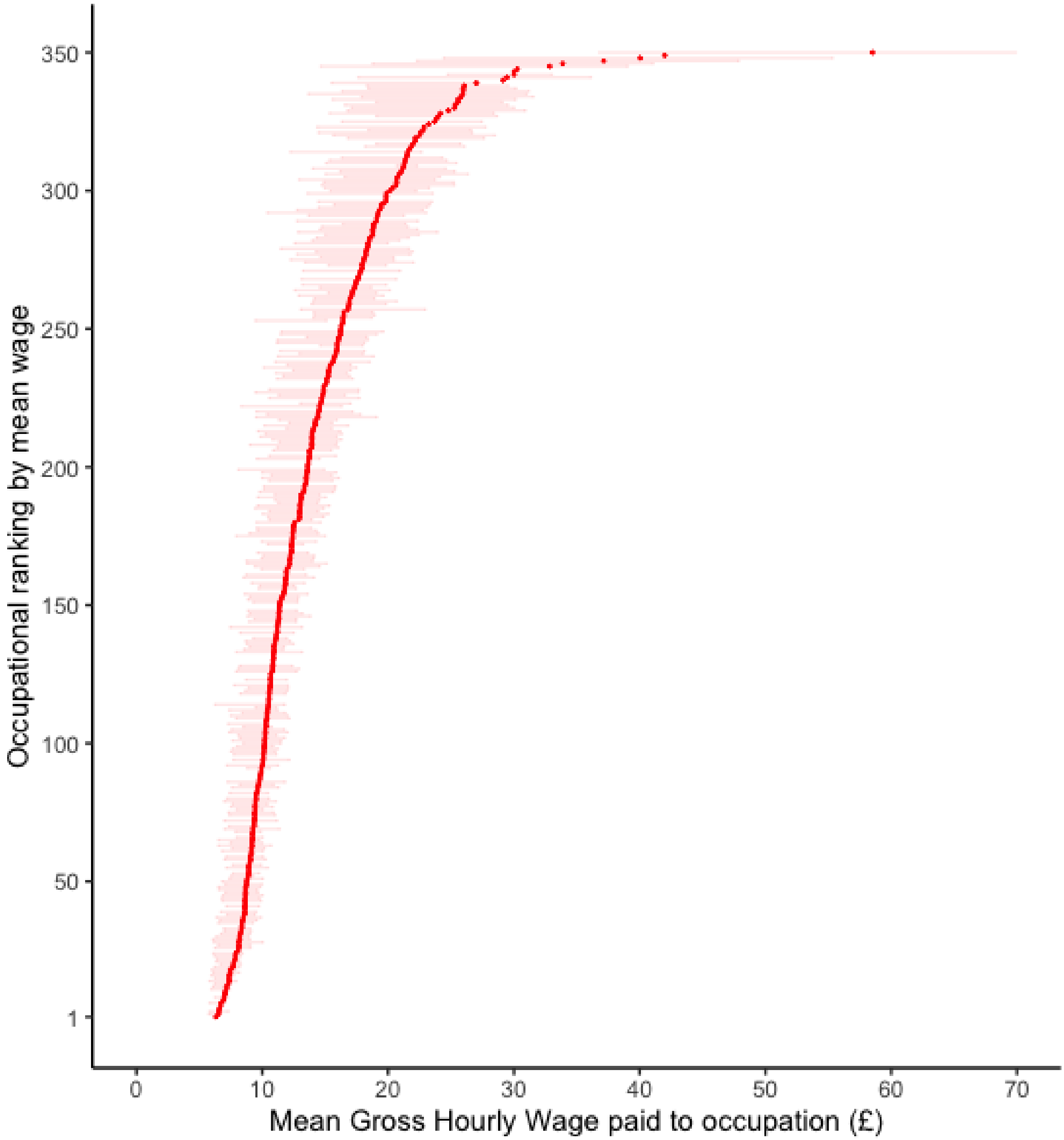

Notes: Individual points represent occupations, bars represent inter-quartile range of wage for that occupation (where data is available). Data is taken from table 14.5a (Hourly pay – Gross 2010) of ASHE 2010.

Figure S2: Distribution of Occupational Wage

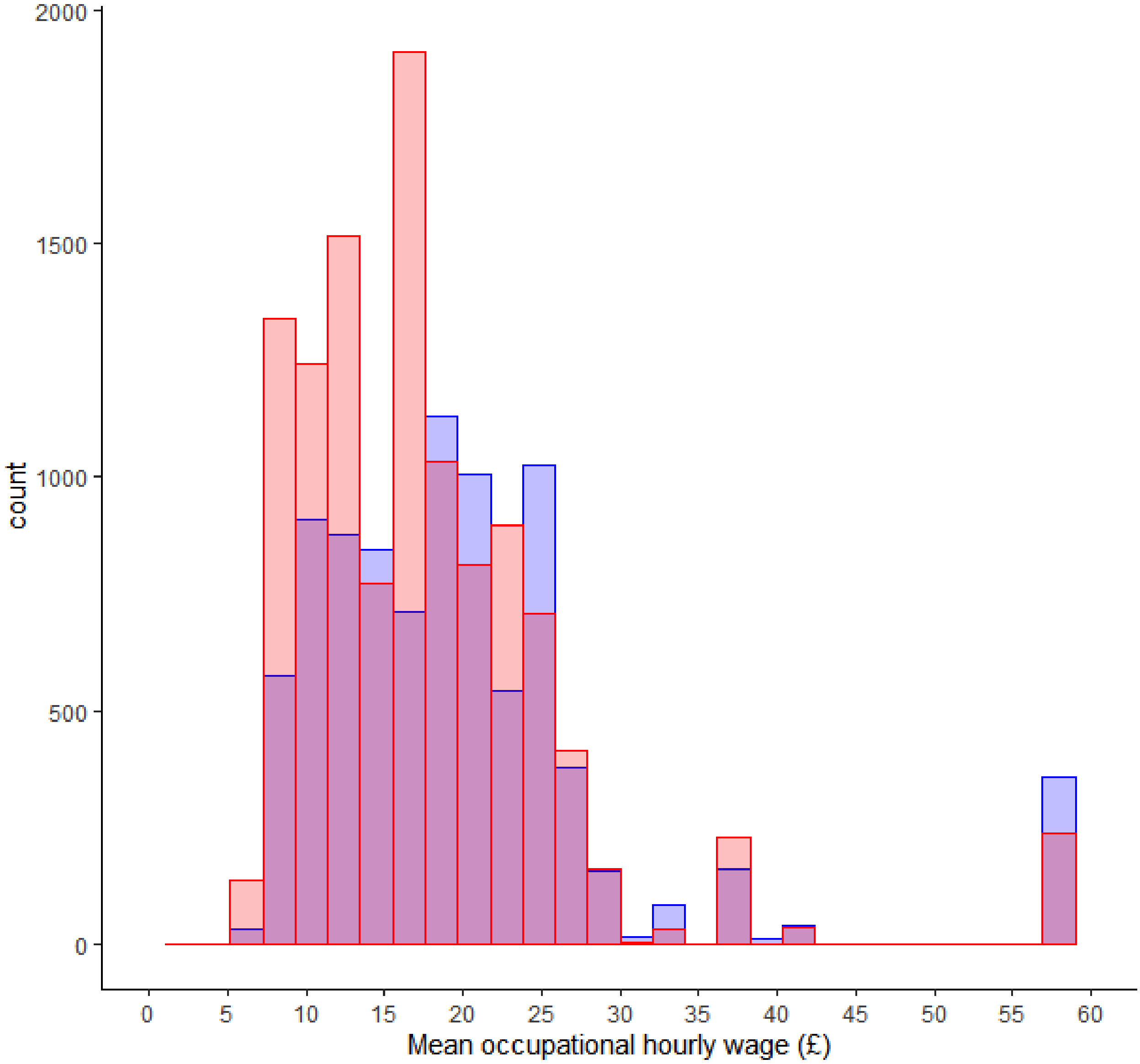

Notes: Graph is a histogram showing distribution of occupational wage in our UK Biobank sample. Red represents women, blue represents men.
